# Phosphorylation of androgen receptors at serine 515 is a potential prognostic marker for triple negative breast cancer

**DOI:** 10.18632/oncotarget.16420

**Published:** 2017-03-21

**Authors:** Antonia K. Roseweir, Pamela McCall, Alison Scott, Benjamin Liew, Zhi Lim, Elizabeth A. Mallon, Joanne Edwards

**Affiliations:** ^1^ Unit of Experimental Therapeutics, Institute of Cancer Sciences, University of Glasgow, Garscube Estate, Glasgow, United Kingdom; ^2^ Academic Unit of Surgery, School of Medicine, University of Glasgow, Royal Infirmary, Glasgow, United Kingdom; ^3^ Department of Pathology, Southern General Hospital, Glasgow, United Kingdom

**Keywords:** androgen receptor, phosphorylation, MAPK, breast cancer, triple negative

## Abstract

1.7 million cases of breast cancer are diagnosed every year with 522,000 deaths. Molecular classifications of breast cancer have resulted in improved treatments. However, treatments for triple negative breast cancer (TNBC) are lacking. Analysis of molecular targets for TNBC is a priority. One potential candidate is androgen receptor (AR) phosphorylation. This study assessed the role of AR phosphorylation at ser81/ser515 and their two upstream effectors, cyclin-dependent kinase 1 (pCDK1) and extracellular-regulated kinase 1/2 (pERK1/2) in 332 ductal breast cancer patients by immunohistochemistry.

pERK1/2 combined with AR-515 associated with improved cancer-specific survival (CSS, *p* = 0.038), decreased size (*p* = 0.001), invasive grade (*p* < 0.001), necrosis (*p* = 0.003), b-lymphocytes (*p* = 0.020), molecular subtype (*p* < 0.001) and estrogen receptor (ER)/progesterone receptor (PR)-status (*p* < 0.001). The cohort was therefore stratified into ER+ve and ER-ve patients. In ER+ve tumours, pERK1/2 combined with AR-515 associated with improved CSS (*p* = 0.038), smaller size (*p* = 0.004), invasive grade (*p* = 0.001), decreased b-lymphocytes (*p* = 0.013) and increased plasma cells (*p* = 0.048). In contrast, in TNBC patients, phosphorylation of AR-515 associated with poorer CSS (*p* = 0.007). pERK1/2 combined with AR-515 associated with decreased inflammation (*p* = 0.003), increased tumour stroma (*p* = 0.003) and tumour budding (*p* = 0.011), with trends towards decrease CSS (*p* = 0.065) and macrophage levels (*p* = 0.093).

In Conclusions, AR-515 may be an important regulator of inflammation in breast cancer potential via ERK1/2 phosphorylation. AR-515 is a potential prognostic marker and therapeutic target for TNBC.

## INTRODUCTION

Approximately 1.7 million cases of breast cancer are diagnosed every year. About 522,000 die of breast cancer yearly making it the 5th most common cause of cancer death globally [1]. A great effort is being channelled to improve the understanding of the pathogenesis of this disease as breast cancer is highly heterogeneous with an increasing number of sub-classifications being made [2–4]. In the past 2 decades, 4 molecular classifications of breast cancer have been extensively studied (Luminal A, Luminal B, HER2-type and Triple negative breast cancer (TNBC)) that have resulted in improved treatments [5, 6].

The majority of breast cancers comprise of luminal A and luminal B subtypes. These subtypes display oestrogen (ER) and/or progesterone receptors (PR) rendering these cancer cells susceptible to endocrine therapies e.g. anti-oestrogen therapies (Tamoxifen) [7, 8]. In HER2 positive breast cancer, the presence of HER2, a tyrosine kinase receptor that is part of the EGFR family, enables targeted therapeutics (Trastuzumab, Pertuzumab) to be effective [9, 10]. However, about 15% of breast cancer cases are triple receptor negative lacking expression of ER, PR or HER2 [11].

It has been shown that TNBC patients displaying pathological complete response (pCR) had better outcomes in terms of progression-free survival than patients who had not achieved pCR [12]. pCR is defined as an absence of any residual invasive breast cancer in the breast and axillary lymph nodes upon completion of chemotherapy [13]. Hence, there is an increasing need for targeted therapies to achieve pCR for TNBC cases. Current pathways that are being studied for this purpose include EGFR, aB-crystallin and androgen receptors (AR) [14–16].

Although numerous studies on AR have been published, the exact role of AR as tumour-promoting or anti-tumorigenic remains undecided. It is known that AR signalling plays a role in regulating cancer cell proliferation and apoptosis [17–19]. AR activation by the binding of androgens such as DHT to AR displaces HSP90 (heat shock protein 90), a chaperone protein to allow the dimerization of AR. The DHT–AR dimer complex then translocates to the nucleus to trigger the activation of the genetic machinery, stimulating cell proliferation. During this process, post-translational modifications such as phosphorylation are triggered at multiple sites within AR. It is now thought that it is these phosphorylation events that fully activate the AR and modulate its different cellular functions [20].

It was also reported that when Tamoxifen-resistant breast cancer cells that expressed high levels of AR were exposed to bicalutamide (anti-androgen), cell growth was shown to decrease as well as reversing Tamoxifen resistance [21], indicating that AR expression may be a mechanism of breast cancer cell adaptation to stimulate cell proliferation in an anti-oestrogen environment. A study by Ayca Gucalp et al. observed that bicalutamide in ER negative and AR positive breast cancer demonstrated improved progression-free survival [22], lending further support that AR or its signalling pathways may be a tangible route for treating patients in the ER negative subgroup.

The MAPK/ERK signalling cascade is heavily involved in the regulation of cancer cell proliferation, growth and differentiation. The stimulation of this pathway leads to the activation of a cascade of kinases (e.g. Ras, Raf, MEK), which ultimately leads to the activation of ERK1/2, which is associated with poor prognosis in breast cancer [23]. One function of ERK1/2 is to phosphorylate AR on ser-515. This is an example of ligand-independent activation via phosphorylation of AR, which is thought to be one of the main mechanisms of AR activation in cancer [24].

In contrast, AR phosphorylation at ser-81 is the prototypical site for dihydrotestosterone (DHT) activation, which is thought to be facilitated by activated Cdk1, as observed by Chen et al where Cdk1 inhibitors decreased ser-81 phosphorylation of AR in LNCaP cells and similarly decreased AR protein expression and transcriptional activity. Whereas transfected Cdk1 stimulated AR phosphorylation at ser-81 and increased AR protein expression [25]. This suggestion has been supported by another study by Yulia et al where *in vitro* kinase assays demonstrated an increase in ser-308 phosphorylation by CDK1 is responsible for Serine–81 phosphorylation [20]. Cdk1 phosphorylation of AR-81 has also been associated with decreased survival in prostate cancer patients [26].

This study aims to further understand the role of androgens and AR activation in breast cancer by determining its effect on patient survival. This is followed by the determination of the role of upstream pathways (i.e. Cdk1 and ERK1/2 pathways) on AR activation and their effects on cancer specific survival (CSS). We aim to confirm that the phosphorylation of AR at either ser-515 or ser-81 is a surrogate for AR activation and assess if the pathways responsible for this phosphorylation are potential targets for therapeutic intervention.

## RESULTS

### Phosphorylation of AR-515 associates with improved cancer-specific survival in ductal breast cancer patients

**Figure 1:**
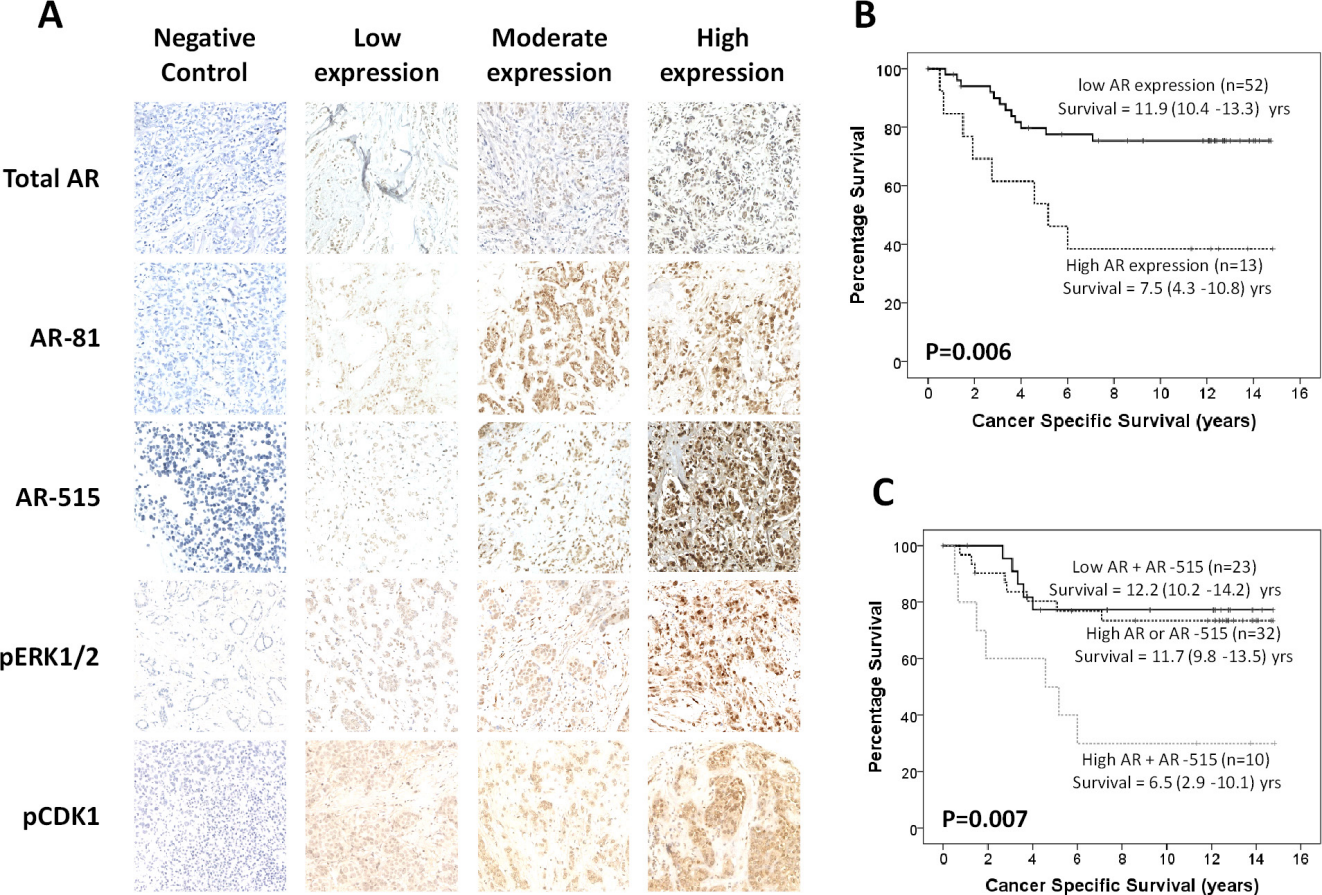
AR-515 is associated with poorer patient survival in triple negative breast cancer (**A**) Representative immunohistochemical images for AR, AR-81, AR-515, pCDK1 and pERK1/2. Negative, low, moderate and high nuclear staining is shown for each protein. Cytoplasmic staining can also be observed for some proteins but was not quantified as it represents the inactive form. (**B**) Kaplan-Meier curve showing that high total AR expression associates with poorer CSS in TNBC patients. (**C**) Kaplan-Meier curve showing that when total AR and AR-515 are combined, high expression of both proteins is associated with poorer CSS in TNBC patients.

Only patients with staining for AR, AR-81 and AR-515 were included in the analysis (*n* = 332, Figure 1A). The majority of the patients (71%) were above 50 years old, had small tumour size ≤ 20 mm (58%), had grade II and III tumours (80%) and no involved lymph nodes (53%). ER positive tumours (69%), PR negative tumours (52%) and HER2 negative tumours (82%) formed the majority of tumours, with 20% of cases being triple negative. Overall, 119 (36%) had lumpectomy and radiotherapy, and 213 (64%) had mastectomy and radiotherapy. 176 (53%) of the patients received endocrine therapy alone, 62 (19%) of patients received adjuvant chemotherapy only, while 76 (23%) had both. The median survival was 148 months with 69 deaths from breast cancer and 68 deaths from non-cancer related causes.

The relationship between nuclear AR activation and CSS is shown in Table 1. Only nuclear expression was investigated as this represents the active form of the protein. High levels of nuclear AR activation via phosphorylation of serine 81 (AR-81) or serine 515 (AR-515) were identified in 155 (47%) and 167 (50%) patients, respectively. Neither AR-81 (*p* = 0.842) nor AR-515 (*p* = 0.563) were associated with CSS alone. Therefore, upstream effectors of AR phosphorylation were investigated. AR-81 significantly correlated with nuclear CDK1 (*p* < 0.001) and AR-515 correlated with nuclear pERK1/2 (*p* < 0.001) as expected. However, AR-81 still showed no associations even when combined with CDK1 in the nucleus (*p* = 0.344). In contrast, AR-515 was significantly associated with improved CSS when combined with ERK1/2 phosphorylation in the nucleus (pERK1/2, *p* = 0.032). When nuclear pERK1/2 was assessed alone it was also significantly associated with improved CSS (*p* = 0.004). Therefore, further analysis was confined to nuclear pERK1/2 and AR-515.

**Table 1:** Relationship between ligand-independent AR phosphorylation and cancer-specific survival in patients with invasive ductal breast cancer (*n* = 332)

	*N* (%)	10yr-CSS %(SE)	Univariate HR(95% CI)	*P*
**Total AR expression**				
** Low expression**	167 (50)	79 (3)	1.04 (0.65–1.67)	0.869
** High expression**	165 (50)	80 (3)		
**AR-81 phosphorylation**				
** Low expression**	177 (53)	79 (3)	0.95 (0.59–1.53)	0.842
** High expression**	155 (47)	78 (4)		
**AR-515 phosphorylation**				
**Low expression**	165 (50)	78 (3)	0.87 (0.54–1.40)	0.563
**High expression**	167 (50)	79 (3)		
**CDK1 phosphorylation (*n*** **= 227)**				
** Low expression**	118 (52)	76 (4)	1.00 (0.57–1.74)	0.985
** High expression**	109 (48)	77 (4)		
**ERK1/2 phosphorylation**				
**Low expression**	151 (45)	72 (4)	0.49 (0.30–0.80)	**0.004**
**High expression**	181 (55)	84 (3)		
**Combined AR + AR-81**				
**Low AR + AR-81**	91 (27)	80 (4)	1.00 (0.72–1.39)	0.851
**High AR or AR-81**	162 (49)	77 (3)		
**High AR + AR-81**	79 (24)	79 (5)		
**Combined AR + AR-515**				
**Low AR + AR-515**	95 (29)	78 (4)	0.96 (0.70–1.31)	0.903
**High AR or AR-515**	142 (42)	79 (4)		
**High AR + AR-515**	95 (29)	79 (5)		
**Combined pCDK1 + AR-81 (*n*** **= 227)**				
**Low pCDK1 + AR-81**	68 (30)	78 (6)	0.88 (0.60–1.28)	0.344
**High pCDK1 or AR-81**	101 (44)	72 (5)		
**High pCDK1 + AR-81**	58 (26)	83 (5)		
**Combined pERK1/2 + AR-515**				
**Low pERK + AR-515**	94 (28)	77 (5)	0.72 (0.53–0.98)	**0.032**
**High pERK or AR-515**	128 (39)	73 (4)		
**High pERK + AR-515**	110 (33)	88 (3)		

The relationship between pERK1/2, AR-515, clinicopathological characteristics, and local inflammatory response is presented in Table 2. pERK1/2 was significantly associated with smaller size (*p* = 0.001), lower grade (*p* < 0.001), ER/PR+ve tumours (*p* < 0.001), molecular subtype (*p* < 0.001), lower necrosis (*p* = 0.003), weak Klintrup-Makinen (KM) grade (*p* = 0.004) and lower b-lymphocyte levels (*p* = 0.020). There was also a trend towards decreased HER2 (*p* = 0.096) and lower lymphovascular invasion (*p* = 0.099). When pERK1/2 was combined with AR-515 significant associations were still seen with smaller size (*p* = 0.017), lower grade (*p* < 0.001), ER+ve tumours (*p* = 0.013), PR+ve tumours (*p* = 0.008), decreased HER2 (*p* = 0.041), molecular subtype (*p* < 0.001), lower necrosis (*p* = 0.006), weak KM grade (*p* = 0.005) and lower b-lymphocytes (*p* = 0.023). Significant associations were now also seen with decreased proliferation (*p* = 0.009), lymphovascular invasion (*p* = 0.017) and increased tumour budding (*p* = 0.047).

**Table 2:** Relationship between clinico-pathological characteristics and ERK1/2 plus AR-515 in patients with invasive ductal breast cancer (*n* = 332)

	pERK1/2			pERK1/2 + AR-515			
	**Low(*n* = 150)**	**High(*n* = 181)**	***P***	**Low pERK + AR-515(*n* = 93)**	**High pERK or AR-515(*n* = 128)**	**High pERK + AR-515(*n* = 110)**	***P***
**Clinicopathological Characteristics**							
**Age**			0.544				0.442
** ≤ 50 years**	46 (31)	50 (28)		29 (31)	32 (25)	35 (31)	
** > 50 years**	104 (69)	131 (72)		64 (69)	96 (75)	75 (69)	
**Size**			**0.001**				**0.017**
** ≤ 20 mm**	74 (49)	117 (65)		46 (49)	73 (57)	72 (65)	
** 21–50 mm**	68 (45)	63 (34)		44 (47)	50 (39)	37 (34)	
** > 50 mm**	8 (6)	1 (1)		3 (4)	5 (4)	1 (1)	
**Invasive Grade**			**< 0.001**				**< 0.001**
** I**	14 (9)	51 (28)		10 (10)	20 (16)	35 (32)	
** II**	57 (38)	84 (46)		35 (38)	64 (50)	42 (38)	
** III**	79 (53)	46 (26)		48 (52)	44 (34)	33 (30)	
**Lymph Node Involvement**			0.874				0.779
** No**	81 (54)	96 (53)		53 (57)	63 (49)	60 (55)	
** Yes**	69 (46)	85 (47)		40 (43)	65 (51)	50 (45)	
**ER Status**			**< 0.001**				**0.013**
** No**	68 (45)	35 (19)		35 (38)	44 (34)	24 (22)	
** Yes**	82 (55)	146 (81)		58 (62)	84 (66)	86 (88)	
**PR status**			**< 0.001**				**0.008**
** No**	94 (63)	76 (42)		55 (59)	70 (55)	45 (41)	
** Yes**	56 (37)	105 (58)		38 (41)	58 (45)	65 (59)	
**Her2 status**			0.096				**0.041**
** No**	120 (80)	157 (87)		74 (80)	104 (81)	99 (90)	
** Yes**	30 (20)	24 (13)		19 (20)	24 (19)	11 (10)	
**Molecular Subtype**			**< 0.001**				**< 0.001**
** Luminal A**	49 (33)	108 (60)		30 (32)	61 (48)	67 (61)	
** Luminal B**	36 (24)	41 (23)		32 (34)	24 (19)	22 (20)	
** TNBC**	44 (29)	23 (13)		21 (23)	27 (21)	18 (16)	
** Her2+**	21 (14)	9 (4)		10 (11)	16 (13)	3 (3)	
**Tumour Necrosis**			**0.003**				**0.006**
** Low**	61 (41)	103 (57)		40 (43)	56 (44)	68 (62)	
** High**	89 (59)	78 (43)		53 (57)	72 (56)	42 (38)	
**Ki67 proliferation index**			0.177				**0.009**
** Low**	107 (71)	140 (77)		59 (63)	100 (78)	88 (80)	
** High**	43 (29)	41 (23)		34 (37)	28 (22)	22 (20)	
**Lymphatic Invasion**			0.961				0.859
** No**	99 (66)	119 (66)		64 (69)	80 (62)	74 (67)	
** Yes**	51 (34)	62 (34)		29 (31)	48 (38)	36 (33)	
**Lymphovascular Invasion**			0.099				**0.017**
** No**	127 (85)	164 (91)		76 (82)	113 (88)	102 (93)	
** Yes**	23 (15)	17 (9)		17 (18)	15 (12)	8 (7)	
**Inflammatory Infiltrate**							
**Klintrup Makinen Grade**			**0.004**				**0.005**
** Weak**	103 (69)	149 (82)		64 (69)	94 (73)	94 (85)	
** Strong**	47 (31)	32 (18)		29 (31)	34 (27)	16 (15)	
**CD68+ macrophages**			0.776				0.228
** Low**	31 (21)	32 (17)		13 (14)	31 (24)	19 (17)	
** Moderate**	53 (35)	79 (44)		38 (41)	42 (33)	52 (47)	
** High**	66 (44)	70 (39)		42 (45)	55 (47)	39 (36)	
**CD4+ T-lymphocytes**			0.156				0.413
** Low**	54 (36)	82 (45)		33 (35)	54 (42)	49 (44)	
** Moderate**	37 (25)	36 (20)		28 (30)	21 (16)	24 (22)	
** High**	59 (39)	63 (35)		32 (35)	53 (41)	37 (34)	
**CD8+ cytotoxic T-cells**			0.458				0.452
** Low**	39 (26)	51 (28)		24 (25)	33 (26)	33 (30)	
** Moderate**	54 (36)	69 (38)		35(38)	47 (37)	41 (37)	
** High**	57 (38)	61 (34)		34 (37)	48 (37)	36 (33)	
**CD20+ B-lymphocytes**			**0.020**				**0.023**
** Low**	65 (43)	100 (55)		42(45)	58 (45)	65 (59)	
** Moderate**	24 (16)	28 (15)		12 (13)	25 (20)	15 (14)	
** High**	61 (41)	53 (30)		39 (42)	45 (35)	30 (27)	
**CD138+ plasma cells**			0.225				0.111
** Low**	78 (52)	78 (43)		51 (55)	60 (47)	45 (41)	
** Moderate**	19 (13)	33 (18)		13 (14)	16 (13)	23 (21)	
** High**	53 (35)	70 (39)		29 (31)	52 (40)	42 (38)	
**Tumour Stroma Percentage**			0.700				0.134
** Low**	100 (66)	117 (65)		64 (69)	88 (69)	65 (59)	
** High**	50 (34)	64 (35)		29 (31)	40 (31)	45 (41)	
** Tumour Budding**			0.532				**0.047**
** Low**	97 (65)	111 (61)		65 (70)	81 (63)	62 (56)	
** High**	53 (35)	70 (39)		28 (30)	47 (37)	48 (44)	
**Angiogenesis (*n* = 321)**			0.586				0.564
** Low**	46 (31)	55 (31)		29 (32)	46 (36)	26 (25)	
** Moderate**	46 (31)	62 (36)		28 (31)	40 (32)	40 (38)	
** High**	55 (38)	57 (33)		34 (37)	40 (32)	38 (37)	

### AR-515 associates with improved cancer-specific survival in ER+ve breast cancer patients

As pERK1/2 plus AR-515 associated with molecular subtype, we firstly stratified patients into ER+ve and ER-ve tumours (Supplementary Table 1). pERK1/2 was associated with improved CSS only in ER+ve tumours (*p* = 0.008). As pERK1/2 and AR-515 strongly correlated in ER+ve patients (*p* < 0.001) they were combined. However, when combined the survival effect was weakened (*p* = 0.038), suggesting that pERK can also act independently of AR-515 in ER+ve breast cancers. When ER+ve patients where stratified further, there was no difference in CSS between luminal A and luminal B subtypes for pERK1/2 alone or combined with AR-515 therefore further analysis was carried out in all ER+ve tumours (*n* = 228).

The relationship between pERK1/2, AR-515, clinicopathological characteristics, and local inflammatory response in ER+ve tumours is presented in Table 3. pERK1/2 was significantly associated with smaller size (*p* = 0.004), lower grade (*p* = 0.001), molecular subtype (*p* = 0.024), decreased proliferation (*p* = 0.006), lower b-lymphocytes (*p* = 0.013) and increased plasma cells (*p* = 0.048). There was also a trend towards decreased necrosis (*p* = 0.096). When pERK1/2 was combined with AR-515 significant associations where still seen with lower grade (*p* = 0.002), molecular subtype (*p* = 0.001), decreased proliferation (*p* = 0.001), lower b-lymphocytes (*p* = 0.006) and increased plasma cells (*p* = 0.023). Significant associations were now also seen with decreased necrosis (*p* = 0.009) and lymphovascular invasion (*p* = 0.029).

**Table 3:** Relationship between clinico-pathological characteristics and AR-515 in patients with ER + ve invasive ductal breast cancer (*n* = 228)

	pERK1/2			pERK1/2 + AR-515			
	Low(*n* = 150)	High(*n* = 181)	*P*	Low pERK + AR-515(*n* = 93)	High pERK or AR-515(*n* = 128)	High pERK + AR-515(*n* = 110)	*P*
**Clinicopathological Characteristics**							
**Age**			0.544				0.442
** ≤ 50 years**	46 (31)	50 (28)		29 (31)	32 (25)	35 (31)	
** > 50 years**	104 (69)	131 (72)		64 (69)	96 (75)	75 (69)	
**Size**			**0.001**				**0.017**
** ≤ 20 mm**	74 (49)	117 (65)		46 (49)	73 (57)	72 (65)	
** 21–50 mm**	68 (45)	63 (34)		44 (47)	50 (39)	37 (34)	
** > 50 mm**	8 (6)	1 (1)		3 (4)	5 (4)	1 (1)	
**Invasive Grade**			**< 0.001**				**< 0.001**
** I**	14 (9)	51 (28)		10 (10)	20 (16)	35 (32)	
** II**	57 (38)	84 (46)		35 (38)	64 (50)	42 (38)	
** III**	79 (53)	46 (26)		48 (52)	44 (34)	33 (30)	
**Lymph Node Involvement**			0.874				0.779
** No**	81 (54)	96 (53)		53 (57)	63 (49)	60 (55)	
** Yes**	69 (46)	85 (47)		40 (43)	65 (51)	50 (45)	
**ER Status**			**< 0.001**				**0.013**
** No**	68 (45)	35 (19)		35 (38)	44 (34)	24 (22)	
** Yes**	82 (55)	146 (81)		58 (62)	84 (66)	86 (88)	
**PR status**			**< 0.001**				**0.008**
** No**	94 (63)	76 (42)		55 (59)	70 (55)	45 (41)	
** Yes**	56 (37)	105 (58)		38 (41)	58 (45)	65 (59)	
**Her2 status**			0.096				**0.041**
** No**	120 (80)	157 (87)		74 (80)	104 (81)	99 (90)	
** Yes**	30 (20)	24 (13)		19 (20)	24 (19)	11 (10)	
**Molecular Subtype**			**< 0.001**				**< 0.001**
** Luminal A**	49 (33)	108 (60)		30 (32)	61 (48)	67 (61)	
** Luminal B**	36 (24)	41 (23)		32 (34)	24 (19)	22 (20)	
** TNBC**	44 (29)	23 (13)		21 (23)	27 (21)	18 (16)	
** Her2+**	21 (14)	9 (4)		10 (11)	16 (13)	3 (3)	
**Tumour Necrosis**			**0.003**				**0.006**
** Low**	61 (41)	103 (57)		40 (43)	56 (44)	68 (62)	
** High**	89 (59)	78 (43)		53 (57)	72 (56)	42 (38)	
**Ki67 proliferation index**			0.177				**0.009**
** Low**	107 (71)	140 (77)		59 (63)	100 (78)	88 (80)	
** High**	43 (29)	41 (23)		34 (37)	28 (22)	22 (20)	
**Lymphatic Invasion**			0.961				0.859
** No**	99 (66)	119 (66)		64 (69)	80 (62)	74 (67)	
** Yes**	51 (34)	62 (34)		29 (31)	48 (38)	36 (33)	
**Lymphovascular Invasion**			0.099				**0.017**
** No**	127 (85)	164 (91)		76 (82)	113 (88)	102 (93)	
** Yes**	23 (15)	17 (9)		17 (18)	15 (12)	8 (7)	
**Inflammatory Infiltrate**							
**Klintrup Makinen Grade**			**0.004**				**0.005**
** Weak**	103 (69)	149 (82)		64 (69)	94 (73)	94 (85)	
** Strong**	47 (31)	32 (18)		29 (31)	34 (27)	16 (15)	
**CD68+ macrophages**			0.776				0.228
** Low**	31 (21)	32 (17)		13 (14)	31 (24)	19 (17)	
** Moderate**	53 (35)	79 (44)		38 (41)	42 (33)	52 (47)	
** High**	66 (44)	70 (39)		42 (45)	55 (47)	39 (36)	
**CD4+ T-lymphocytes**			0.156				0.413
** Low**	54 (36)	82 (45)		33 (35)	54 (42)	49 (44)	
** Moderate**	37 (25)	36 (20)		28 (30)	21 (16)	24 (22)	
** High**	59 (39)	63 (35)		32 (35)	53 (41)	37 (34)	
**CD8+ cytotoxic T-cells**			0.458				0.452
** Low**	39 (26)	51 (28)		24 (25)	33 (26)	33 (30)	
** Moderate**	54 (36)	69 (38)		35(38)	47 (37)	41 (37)	
** High**	57 (38)	61 (34)		34 (37)	48 (37)	36 (33)	
**CD20+ B-lymphocytes**			**0.020**				**0.023**
** Low**	65 (43)	100 (55)		42(45)	58 (45)	65 (59)	
** Moderate**	24 (16)	28 (15)		12 (13)	25 (20)	15 (14)	
** High**	61 (41)	53 (30)		39 (42)	45 (35)	30 (27)	
**CD138+ plasma cells**			0.225				0.111
** Low**	78 (52)	78 (43)		51 (55)	60 (47)	45 (41)	
** Moderate**	19 (13)	33 (18)		13 (14)	16 (13)	23 (21)	
** High**	53 (35)	70 (39)		29 (31)	52 (40)	42 (38)	
**Tumour Stroma Percentage**			0.700				0.134
** Low**	100 (66)	117 (65)		64 (69)	88 (69)	65 (59)	
** High**	50 (34)	64 (35)		29 (31)	40 (31)	45 (41)	
** Tumour Budding**			0.532				**0.047**
** Low**	97 (65)	111 (61)		65 (70)	81 (63)	62 (56)	
** High**	53 (35)	70 (39)		28 (30)	47 (37)	48 (44)	
**Angiogenesis (*n* = 321)**			0.586				0.564
** Low**	46 (31)	55 (31)		29 (32)	46 (36)	26 (25)	
** Moderate**	46 (31)	62 (36)		28 (31)	40 (32)	40 (38)	
** High**	55 (38)	57 (33)		34 (37)	40 (32)	38 (37)	

### AR phosphorylation at Ser515 associates with poorer patient survival in TNBC

It was observed in patients with ER-ve breast cancer that high pERK1/2 plus AR-515 had a trend towards an effect on survival rate. It was hypothesised that the loss of significance and weaker correlation between pERK1/2 and AR-515 in ER-ve patients (*p* = 0.023) may be due to differences between the two ER-ve subtypes. Therefore, ER-ve patients were stratified into HER2-type and TNBC to further investigate these effects (Supplementary Table 2). In patients with TNBC, the correlation between pERK1/2 and AR-515 was strengthened (*p* = 0.010), however no significant effect on CSS was observed for pERK1/2 alone (*p* = 0.534) and only a trend towards poorer outcome was seen when combined with AR-515 (*p* = 0.065). However, high levels of AR-515 combined with total AR, representing full AR activation, associated with a significantly poorer outcome (*p* = 0.007, Figure 1A), and a similar effect was also seen when total AR was considered alone (*p* = 0.006, Figure 1B). In contrast, in patients with HER2-type cancers the correlation between pERK1/2 and AR-515 was lost (*p* = 0.949) and no significant associations were seen in these patients. However, there was a trend towards improved patient survival with AR-515 phosphorylation (*p* = 0.056) alone and when combined with pERK1/2 (*p* = 0.076), confirming our hypothesis that these two ER-ve subtypes respond differently to AR-515 phosphorylation.

Due to the detrimental effect of AR-515 phosphorylation in TNBC, associations with clinicopathological characteristics were assessed (Table 4). Full activation of the AR receptor as shown by AR-515 plus total AR significantly associated with increased lymphovascular invasion (*p* = 0.033), lower KM grade (*p* = 0.004), increased tumour-stroma percentage (TSP) (*p* = 0.033) and increased tumour budding (*p* = 0.007). When combined with pERK1/2 associations were still seen with lower KM grade (*p* = 0.003), increased TSP (*p* = 0.003) and increased tumour budding (*p* = 0.011). Trends towards association were also seen for increased age (*p* = 0.075) and decreased macrophages (*p* = 0.093).

**Table 4:** Relationship between clinico-pathological characteristics, pERK1/2 and AR-515 in patients with TNBC (*n* = 65)

	AR + AR-515				pERK1/2 + AR-515			
	**Low AR + AR-515 (*n* = 21)**	**High AR or AR-515** (***n* = 27)**	**High AR + AR-515** (***n* = 17)**	***P***	**Low pERK + AR-515** (***n* = 23)**	**High pERK or AR-515** (***n* = 32)**	**High pERK + AR-515** (***n* = 10)**	***P***
**Clinicopathological Characteristics**								
**Age** ** ≤ 50 years** ** > 50 years**	11 (48) 12 (52)	12 (37) 20 (63)	3 (30) 7 (70)	0.304	12 (57) 9 (43)	9 (33) 18 (67)	5 (29) 12 (71)	0.075
**Size** ** ≤ 20 mm** ** 21–50 mm** ** > 50 mm**	6 (26) 17 (74) 0 (0)	20 (63) 11 (34) 1 (3)	3 (30) 6 (60) 1 (10)	0.648	6 (29) 15 (71) 0 (0)	12 (44) 13 (48) 2 (8)	11 (64) 6 (36) 0 (0)	0.051
**Invasive Grade** ** I** ** II** ** III**	2 (9) 3 (13) 18 (78)	1 (3) 7 (22) 24 (75)	0 (0) 2 (20) 8 (80)	0.641	1 (5) 2 (10) 18 (85)	1 (4) 6 (22) 20 (74)	1 (6) 4 (24) 12 (70)	0.353
**Lymph Node Involvement** ** No** ** Yes**	14 (62) 9 (39)	19 (59) 13 (41)	3 (30) 7 (70)	0.169	14 (66) 7 (34)	13 (48) 14 (52)	9 (53) 8 (47)	0.366
**Tumour Necrosis** ** Low** ** High**	5 (22) 18 (78)	11 (34) 21 (66)	1 (10) 9 (90)	0.806	5 (24) 16 (76)	6 (22) 21 (78)	6 (35) 11 (65)	0.452
**Ki67 proliferation index** ** Low** ** High**	17 (74) 6 (26)	27 (84) 5 (16)	8 (80) 2 (20)	0.480	17 (81) 4 (19)	22 (81) 5 (19)	13 (76) 4 (24)	0.744
**Lymphatic Invasion** ** No** ** Yes**	15 (65) 8 (35)	20 (62) 12 (38)	4 (40) 6 (60)	0.240	15 (71) 6 (29)	16 (59) 11 (41)	8 (53) 9 (47)	0.129
**Lymphovascular Invasion** ** No** ** Yes**	19 (83) 4 (17)	28 (87) 4 (13)	5 (50) 5 (50)	**0.033**	17 (81) 4 (19)	21 (78) 6 (22)	14 (82) 3 (18)	0.936
**Inflammatory Infiltrate**								
**Klintrup Makinen Grade** ** Weak** ** Strong**	6 (26) 17 (74)	22 (69) 10 (31)	7 (70) 3 (30)	**0.004**	5 (24) 16 (76)	18 (67) 9 (33)	12 (70) 5 (30)	**0.003**
**CD68+ macrophages** ** Low** ** Moderate** ** High**	3 (13) 8 (35) 12 (52)	10 (31) 6 (19) 16 (50)	3 (30) 5 (50) 2 (20)	0.111	2 (10) 7 (34) 12 (56)	11 (41) 5 (18) 11 (41)	3 (18) 7 (41) 7 (41)	0.093
**CD4+ T-lymphocytes** ** Low** ** Moderate** ** High**	5 (22) 8 (35) 10 (43)	6 (19) 6 (19) 20 (62)	5 (50) 1 (10) 4 (40)	0.604	3 (14) 7 (34) 11 (52)	10 (37) 4 (15) 13 (48)	3 (18) 4 (24) 10 (58)	0.983
**CD8+ cytotoxic T-cells** ** Low** ** Moderate** ** High**	4 (12) 8 (35) 11 (47)	7 (22) 4 (13) 21 (65)	2 (20) 6 (60) 2 (20)	0.522	4 (19) 6 (29) 12 (52)	7 (26) 7 (26) 13 (48)	3 (17) 5 (30) 9 (53)	0.726
**CD20+ B-lymphocytes** ** Low** ** Moderate** ** High**	8 (35) 3 (13) 12 (52)	9 (28) 5 (16) 18 (56)	2 (20) 2 (20) 6 (60)	0.488	7 (29) 3 (19) 12 (52)	10 (37) 6 (22) 11 (41)	3 (18) 1 (6) 13 (76)	0.231
**CD138+ plasma cells** ** Low** ** Moderate** ** High**	7 (29) 2 (9) 14 (62)	11 (34) 7 (22) 14 (44)	3 (30) 1 (10) 6 (60)	0.779	6 (29) 2 (10) 13 (61)	12 (44) 4 (15) 11 (41)	3 (18) 4 (24) 10 (58)	0.302
**Tumour Stroma Percentage** ** Low** ** High**	21 (91) 2 (9)	19 (59) 13 (41)	7 (70) 3 (30)	**0.033**	19 (90) 2 (10)	20 (74) 7 (26)	8 (53) 9 (47)	**0.003**
**Tumour Budding** ** Low** ** High**	20 (87) 3 (13)	22 (69) 10 (31)	4 (40) 6 (60)	**0.007**	19 (90) 2 (10)	18 (67) 9 (33)	9 (53) 8 (47)	**0.011**
**Angiogenesis** ** Low** ** Moderate** ** High**	10 (51) 4 (12) 9 (39)	10 (31) 6 (19) 16 (50)	2 (20) 3 (30) 5 (50)	0.255	9 (43) 3 (14) 9 (43)	8 (30) 7 (26) 12 (44)	5 (30) 3 (17) 9 (53)	0.413

### Is AR phosphorylation at Ser515 an independent prognostic factor for ER+ve or TNBC?

For ER+ve tumours, since pERK1/2 plus AR-515 had significant associations with improved patient survival, these factors were taken into univariate and multivariate analysis (Table 5). On univariate analysis, size (*p* = 0.002), invasive grade (*p* = 0.013), lymph node involvement (*p* = 0.009), necrosis (*p <* 0.001), proliferation index (*p <* 0.001), lymphatic invasion (*p <* 0.001), cytotoxic t-lymphocytes (*p* = 0.005), TSP (*p* = 0.009), tumour budding (*p* = 0.001) and angiogenesis (*p* = 0.043) were significantly associated with poorer CSS. Lymphovascular invasion (*p* = 0.054) and b-lymphocytes (*p* = 0.064) trended towards significance.

**Table 5:** Univariate and multivariate survival analysis in patients with invasive ductal breast cancer (*n* = 293)

	ER + ve (*n* = 228)				TNBC (*n* = 65)			
	Univariate HR(95% CI)	*P*	Multivariate HR(95% CI)	*P*	Univariate HR(95% CI)	*P*	Multivariate HR(95% CI)	*P*
**Clinicopathological Characteristics**								
**Age (< 50/> 50)**	1.92 (0.80–4.62)	0.143	-	-	2.70 (0.98–7.44)	0.055	-	-
**Size (< 20/20–50/ > 50)**	2.38 (1.36–4.15)	**0.002**	1.58 (0.67–2.89)	0.134	3.23 (1.19–7.78)	0.022	1.20 (0.42–3.40)	0.732
**Invasive Grade (I/II/III)**	1.83 (1.14–2.95)	**0.013**	1.44 (0.83–2.48)	0.193	1.38 (0.54–3.51)	0.502	-	-
**Lymph Node Involvement (No/Yes)**	2.47 (1.25–4.88)	**0.009**	1.48 (0.68–3.20)	0.324	7.16 (2.38–21.5)	< 0.001	5.17 (1.59-16.8)	**0.006**
**Tumour Necrosis (Low/High)**	3.19 (1.75–6.95)	**< 0.001**	2.33 (1.06–5.14)	**0.036**	9.25 (1.34–69.2)	0.030	4.82 (0.57-40.5)	0.148
**Ki67 proliferation index (Low/High)**	3.59 (1.87–6.86)	**< 0.001**	2.59 (1.21–5.53)	**0.014**	0.74 (0.22–2.53)	0.629	-	-
**Lymphatic Invasion (No/Yes)**	3.40 (1.76–6.55)	**< 0.001**	2.17 (0.97–4.86)	0.060	5.27 (1.91–14.5)	0.001	2.61 (0.80-8.53)	0.111
**Lymphovascular Invasion (No/Yes)**	2.25 (0.99–5.12)	0.054	-	-	10.4 (4.13–26.0)	< 0.001	6.04 (1.98-18.4)	**0.002**
**Inflammatory Characteristics**								
**Klintrup-Makinen Grade (Weak/Strong)**	0.71 (0.25–1.99)	0.509	-	-	1.04 (0.43–2.50)	0.938	-	-
**CD68+ macrophages (low/mod/high)**	1.01 (0.64–1.60)	0.961	-	-	0.49 (0.29–0.85)	**0.011**	0.32 (0.16–0.66)	**0.002**
**CD4+ T-lymphocytes (low/mod/high)**	0.78 (0.53–1.41)	0.199	-	-	1.02 (0.60–1.75)	0.933	-	-
**CD8+ cytotoxic T-cells (low/mod/high)**	0.54 (0.35–0.83)	**0.005**	0.44 (0.27–0.72)	**0.001**	0.58 (0.34–0.99)	**0.047**	0.72 (0.36–1.43)	0.346
**CD20+ B-lymphocytes (low/mod/high)**	0.67 (0.43–1.03)	0.064	-	-	1.15 (0.69–1.90)	0.600	-	-
**CD138+ plasma cells (low/mod/high)**	1.11 (0.77–1.60)	0.561	-	-	1.76 (0.99–3.14)	0.055	-	-
**Tumour Stroma Percentage (low/high)**	2.38 (1.25–4.54)	**0.009**	2.13 (1.06–4.27)	**0.033**	2.09 (0.85–5.12)	0.107	-	-
**Tumour Budding (low/high)**	3.07 (1.56–6.03)	**0.001**	1.97 (0.91–4.26)	0.084	1.69 (0.69–4.13)	0.251	-	-
**Angiogenesis (low/high)**	1.55 (1.01–2.37)	**0.043**	1.64 (1.00–2.70)	0.051	1.42 (0.84–2.42)	0.193	-	-
AR Pathway								
**Total AR (low/high)**	1.43 (0.72–2.84)	0.309	-	-	3.31 (1.35–8.11)	**0.006**	1.14 (0.41–3.17)	0.801
**AR-515 (low/high)**	0.78 (0.41–1.51)	0.459	-	-	1.70 (0.65–4.42)	0.274	-	-
**pERK1/2 (low/high)**	0.43 (0.22–0.82)	**0.008**	0.67 (0.43–1.04)	0.073	1.33 (0.54–3.25)	0.534	-	-
**AR+AR-515 (low AR + AR-515/high AR or AR-515/high AR + AR-515)**	1.04 (0.68–1.60)	0.985	-	-	2.19 (1.14–4.20)	**0.007**	0.96 (0.26–3.58)	0.957
**pERK1/2+AR-515 (low pERK + AR-515/high pERK or AR-515/high pERK + AR-515)**	0.64 (0.42–0.98)	**0.038**	0.76 (0.20–2.87)	0.682	1.35 (0.77–2.34)	0.065	-	-

On multivariate survival analysis including pERK1/2 plus AR-515; necrosis (*p* = 0.036), proliferation index (*p* = 0.014), cytotoxic t-lymphocytes (*p* = 0.001), and TSP (*p* = 0.033) were independent prognostic factors, with lymphatic invasion (*p* = 0.060), tumour budding (*p* = 0.084), angiogenesis (*p* = 0.051) and pERK1/2 (*p* = 0.073) showing trends towards independence. However, pERK1/2 plus AR-515 does not appear to be independently prognostic in these patients (*p* = 0.682).

For TNBC, since AR-51 plus total AR had significant associations with poorer patient survival, these factors were taken into univariate and multivariate analysis (Table 7). On univariate analysis, size (*p* = 0.022), lymph node involvement (*p <* 0.001), necrosis (*p* = 0.030), lymphatic invasion (*p* = 0.001), lymphvascular invasion (*p <* 0.001), macrophages (*p* = 0.011) and cytotoxic t-lymphocytes (*p* = 0.047) were significantly associated with poorer CSS. Age (*p* = 0.055) and plasma cells (*p* = 0.055) trended towards significance.

On multivariate survival analysis including AR-515 plus total AR; lymph node involvement (*p* = 0.006), lymphovascular invasion (*p* = 0.002), and macrophages (*p* = 0.002) were independent prognostic factors. However, AR-515 plus total AR (*p* = 0.957) nor AR alone (*p* = 0.801) appear to be independently prognostic in these patients.

## DISCUSSION

This study suggests that effects of activation via phosphorylation of AR at Ser515 are subtype-dependent. In ER+ve and HER2-type patients AR-515 is associated with good prognosis, but in TNBC patients AR-515 is associated with poor prognosis. These effects of AR phosphorylation appear to be associated with inflammation. In ER+ve tumours AR-515 was associated with an increase in b-lymphocytes, therefore combating tumour spread. In contrast, in TNBC tumours AR-515 was associated with a decrease in macrophages, increased proliferation, increased tumour to stroma percentage and invasion. Emphasising the need for a personalised medicine approach, as the TNBC patients could benefit from anti-androgen therapy.

In the current study, AR-515 phosphorylation is associated with decreased Ki67 in ER+ve tumours, confirming previous reports that AR is a good prognostic factor in ER+ve breast cancer due to an anti-proliferative effect [27, 28]. In contrast in TNBC, AR expression is associated with poor prognosis, an increase in proliferation rate, lymphatic invasion and decreased survival [27, 29, 30]. However, current data on the role of AR in breast cancer is controversial with some groups showing a good prognosis for AR-positive TNBC patients [31, 32]. Conversely, other groups have reported similar outcomes to the present study showing a good prognosis for AR/ER-positive patients and a poor prognosis for AR-positive TNBC patients [33, 34]. However, these studies only considered total AR expression, with no analysis of AR phosphorylation and data from the current study suggests that these opposing effects of AR may be due to differential phosphorylation of AR and therefore differential survival effects.

To date little work has been reported on AR phosphorylation in breast cancer, even though this is now thought to be the main post-translational modification needed to fully activate AR [20]. One study observed up-regulation of AR-213 and AR-650 phosphorylation in invasive ductal adenocarcinoma; however patient survival was not investigated [35]. To our knowledge the current study is the first to report AR-515 phosphorylation is associated with poorer patient outcomes in TNBC. This is in line with observations in prostate cancer where AR-515 was associated with decreased CSS and proliferation [26].

ERK1/2 has been proposed to phosphorylate AR-515 in response to EGF stimulation. Ponguta et al. used site directed mutagenesis within the n-terminal region of AR to delineate that AR-515 was the phosphorylation site affected by EGF stimulation [36]. Chia et al. also showed that inhibition of ERK1/2 phosphorylation reduces expression of AR in ER-ve breast cancers. Interestingly, they also showed that AR inhibition leads to down-regulation of ERK1/2 target genes suggesting that AR can regulate ERK1/2 phosphorylation and kinase activity, creating a feedback loop [37]. In the current study, AR and pERK1/2 show a strong correlation and combination of these two proteins strengthens their individual effects, suggesting that ERK1/2 may be responsible for phosphorylation of AR-515 in these patients.

In the current study, AR-515 is present in all breast cancers; however the phenotypic and survival affects are subtype-dependent. In ER+ve breast cancers, AR-515 phosphorylation leads to an improved survival, this may be due to AR-515 increasing the rate of maturation of b-lymphocytes into plasma cells. This is in contrast to a previous study, that suggests total AR can inhibit b-lymphocyte development resulting in suppression of systemic inflammation, however AR phosphorylation sites were not taken into account [38]. It is also interesting that in the current study only pERK1/2 was shown to be an independent prognostic factor in ER+ve tumours, suggesting it may be a driver of this pathway potentially via phosphorylation of AR-515.

In TNBC we observed that phosphorylation of AR-515 leads to poorer survival possibly via down-regulation of inflammation. This has previously been observed in prostate cancer, where DHT treatment and AR activation was associated with decreased local inflammation, inflammatory growth factor production and T-lymphocyte proliferation [39]. This detrimental effect of AR-515 on inflammation and survival, suggests that anti-androgen therapy may be of therapeutic benefit to patients with TNBC. This is in line with current clinical trials were TNBC patients are responding to anti-androgen therapy [22, 40]. Currently, 30% of TNBC patients respond to chemotherapy, but there are no approved targeted treatments for the other 70%. Clinical trials are underway for the use of PARP inhibitors in BRCA1 mutated TNBC and PI3K inhibitors in PTEN mutated TNBC. Anti-androgens are a being trialled as potential treatment for AR-positive patients, which is currently around 10–15% TNBC patients, however using AR phosphorylation as a biomarker rather than total AR expression may increase the frequency of AR in TNBC, as even when AR expression is low, the receptor can still be activated via one of its many phosphorylation sites [20]. It has recently been shown that combining anti-androgens with a PI3K inhibitor increases their sensitivity in TNBC patients [41].

In conclusion, this study demonstrates subtype-specific differences in the effects of AR-515 phosphorylation in breast cancer patients. In ER+ve breast cancers, high phosphorylation of AR-515 conveys a protective effect. This is potentially due to an increased inflammatory response within the tumour when AR-515 is phosphorylated. However, in TNBC patients, phosphorylation of AR-515 associates with a poorer prognosis, possibly due to a down regulation of local inflammation. This difference in survival may be due to different substrates phosphorylating AR-515. In ER+ve patients, AR-515 strongly correlates with pERK1/2, suggesting ERK1/2 may phosphorylate AR-515 in these patients. However, this is not seen in TNBC patients suggesting another kinase is responsible. The data also suggests that AR may be an important regulator of inflammation in breast cancer and maybe a potential prognostic biomarker for TNBC. It also confirms the need to consider phosphorylation of the AR when investigating activation, as differing phosphorylation sites have varied downstream effects. Further investigation is now needed to delineate how AR phosphorylation is influencing inflammation, is it a direct association or is another factor involved. The data for TNBC also needs to be confirmed in a larger dataset as one limitation of this study is the low number of TNBC cases within the cohort.

## MATERIALS AND METHODS

### Patient cohort characteristics

Patients presenting with invasive ductal breast cancer at Glasgow Royal Infirmary, Western Infirmary and Stobhill Hospital between 1995 and 1998 with formalin-fixed paraffin embedded blocks of the primary tumour available for evaluation were studied (*n* = 474). The study was approved by the Research Ethics Committee of the West Glasgow University Hospitals NHS Trust.

Clinicopathological data including age, tumour size, tumour grade, lymph node status, type of surgery and use of adjuvant treatment (chemotherapy, hormonal therapy and/or radiotherapy) were retrieved from the routine reports. Tumour grade was assigned according to the Nottingham Grading System. ER and PR status were assessed on tissue microarrays (TMAs) using immunohistochemistry (IHC) with Dako (Glostrup, Denmark) ER antibody and Leica (Wetzlar, Germany) PR antibody and scored according to the American Society of Clinical Oncology and College of American Pathologists guidelines with a cut-off value of 1% positive tumour nuclei [42]. Her2 status were assessed visually using TMAs as previously described, that is, a score 3 þ is regarded as positive; 2 þ is regarded as equivocal, leading to referral for Her-2 FISH; and 0 and 1 þ are regarded as negative [43].

Full-section haematoxylin and eosin (H&E) slides for the 474 patients were used to score local inflammatory infiltrate according to Klintrup-Makinen (KM) criteria. KM scoring of slides was carried out as previously described. Briefly, tumours were scored on four-point scores based on appearances at the tumour invasive margin. A score of 0 signified that there were no inflammatory cells at tumour's invasive margin; score 1 indicated a mild and patchy inflammatory cells; score 2 denoted a prominent band-like inflammatory reaction at the invasive margin; and score 3 revealed a florid cup-like inflammatory infiltrate at the invasive edge [43, 44]. Individual immune cell types were assessed using IHC staining on TMA sections for macrophages, helper and cytotoxic T-lymphocytes and plasma cells using CD68, CD4, CD8 and CD138 antibodies, respectively [45].

Full-section H&E slides were also used to score the tumour stroma percentage (TSP) as previously reported [46]. Briefly, at 5x magnification, an area representative of the tumour invasive margin was selected, and then a single field of 10x magnification was examined, ensuring that tumour cells were present at all four sides of the image and the area of stroma was calculated as a percentage.

Lymphatic and blood vessel (lymphovascular) invasion were assessed, on 2.5-μm thick sections, using IHC staining with the lymphatic endothelial marker D2-40 (Covance, Monoclonal Antibody, SIG-3730, Princeton, NJ, USA) diluted 1:100 and vascular endothelial marker Factor VIII (Mouse Monoclonal Antibody, NCL-L-Vwf, Leica, Newcastle, UK) diluted 1:100 as previously described [47]. Ki67 proliferation index was assessed by IHC using the median as the cut-off. Tumour budding was evaluated as clusters of 1-5 cancer cells within the tumour microenvironment.

The molecular subtypes were defined as follows: Luminal A: oestrogen (ER) and/or progesterone receptor (PR) positive, Her-2 negative or low proliferative index (< 15%); Luminal B: hormone receptor positive, Her-2 positive or high proliferative index (> 15%); Her-2 subtype: Her-2 positive and hormone receptor negative, any proliferative index; and triple negative: Her-2 negative, hormone receptor negative, any proliferative index.

### Immunohistochemistry

Antibody validation for AR-81, AR-515, pERK1/2 and pCDK1 was carried out as previously described [23, 26]. Immunohistochemistry (IHC) was conducted in triplicate on TMAs for Cdk1 phosphorylated at Thr-161 (pCdk1), phosphorylated ERK1/2 (pERK1/2), total AR and AR phosphorylated at ser-81 (AR-81), or ser-515 (AR-515). Slides were dewaxed in xylene and rehydrated through graded alcohol. For total AR, antigen retrieval was performed in Dako cytomation target retrieval solution (Dako UK Ltd.) in a pre-heated water bath, 99°C, 20 min. pERK1/2 antigen retrieval was performed in pH9 Tris-EDTA buffer (10 mM Trizma Base, 0.25 mM EDTA), 96°C, 20 min. Antigen retrieval for remaining proteins was performed under pressure in Tris-EDTA buffer (5 mM Trizma Base, 1 mM EDTA, pH 8), 5 min. Endogenous peroxidase were blocked with 3% H_2_O_2_. Sections were further blocked using 5% horse serum (10% casein for pERK1/2) in Tris-buffered saline (TBS). Antibodies for pCdk1 (Abcam), pERK1/2 (Cell Signaling Technology, USA), AR-81 (Merk Millipore, USA), AR-515 (Eurogentec Ltd., Southampton, UK) were incubated overnight at 4°C diluted in Dako antibody diluent at 1:150 (Dako UK Ltd.) Total AR antibody (Dako UK Ltd.) was incubated overnight at 4C at 1:4000. Bound antibody complex was visualised using EnVision plus kit (Dako UK Ltd.) followed by 3,3-diaminobenzidine tetrahydrochloride (DAB, Dako UK Ltd.). Nuclei were counterstained with haematoxylin and blued with Scots Tap Water Substitute, dehydrated through graded alcohol and xylene and mounted with Di-N-Butyl Phthalate in xylene (DPX).

TMAs were scanned using the Hamamatsu NanoZoomer (Welwyn Gardens City, Hertfordshire, UK) at × 20 magnification, and visualization was carried out using Slidepath Digital Image Hub, version 4.0.1 (Leica Biosystems, UK).

### Scoring

Only nuclear expression was scored as this represents the active form of each protein. Tissue staining intensity was scored by two blinded independent observers using a weighted histo-score (H-score) method [48, 49]. The H-score was calculated from the formula: (0 × % cells staining negative) + (1 × % cells staining weakly positive) + (2 x % cells staining moderately positive) + (3 × % cells staining strongly positive). The mean H-score from staining conducted in triplicate was used for analysis. Intra-class correlation coefficients (ICCCs) confirmed histo-scoring consistency between observers [49].

### Statistical analysis

Only patients with a score for AR, AR-81 and AR-515 were included in the analysis (*n* = 332). Cut-off values for high or low protein expression were determined by the median of the average values of the intensity of staining. The relationships between variables were assessed using contingency table analysis with the *X*^2^ test for linear trend. Correlations coefficients were analysed using a Spearman's rho. Kaplan– Meier curves with log rank analysis was used to examine the effect of protein expression on CSS. Univariate survival analysis was performed using Cox proportional hazards regression. Variables with *P-value* of < 0.05 were entered into a multivariable model using a backwards conditional method for all patients and triple negative patients. All statistical analyses were two-sided and significance defined as *P-value* < 0.05. All statistical analysis was performed using the SPSS software version 22 (IBM SPSS, Chicago, IL, USA).

## SUPPLEMENTARY MATERIALS FIGURES AND TABLES


