# Supplementary material for: Phosphorylation of androgen receptors at serine 515 is a potential prognostic marker for triple negative breast cancer
Source: Oncotarget. 2017 Mar 21;8(23):37172–85. doi: 10.18632/oncotarget.16420 (PMC5514900; doi:10.18632/oncotarget.16420)
Supplement: Supplementary file 1 [file oncotarget-08-37172-s001.pdf]

Phosphorylation of androgen receptors at serine 515 is a potential prognostic marker for triple negative breast cancer

Supplementary Materials

Supplementary Table 1: Relationship between ligand-independent AR phosphorylation and cancer-specific survival in patients with ER+ve and ER-ve invasive ductal breast cancer

	ER+ve (n = 228)				ER-ve (n = 104)			
	N (%)	10yr-CSS % (SE)	Univariate HR (95% CI)	*P*	*N (%)*	10yr-CSS % (SE)	Univariate HR (95% CI)	*P*
**Total AR expression**								
**Low expression**	89 (39)	87 (4)	1.43 (0.72–2.84)	0.309	78 (75)	69 (5)	1.41 (0.67–2.98)	0.365
**High expression**	139 (61)	82 (4)			26 (22)	62 (10)		
**AR-515 phosphorylation**								
**Low expression**	118 (52)	83 (4)	0.78 (0.41–1.51)	0.459	47 (45)	66 (7)	0.85 (0.43–1.71)	0.656
**High expression**	110 (48)	85 (4)			57 (55)	69 (6)		
**ERK1/2 phosphorylation**								
**Low expression**	82 (36)	77 (5)	0.43 (0.22–0.82)	**0.008**	69 (66)	66 (6)	0.88 (0.41-1.86)	0.728
**High expression**	146 (64)	88 (3)			35 (34)	71 (8)		
**Combined AR + AR-515**								
**Low AR+AR-515**	56 (24)	86 (5)	1.04 (0.68–1.60)	0.985	39 (38)	66 (8)	1.06 (0.65–1.73)	0.721
**High AR or AR-515**	95 (42)	83 (4)			47 (45)	71 (7)		
**High AR + AR-515**	77 (34)	84 (5)			18 (17)	61 (11)		
**Combined pERK1/2 + AR-515**								
**Low pERK1/2+AR-515**	58 (25)	80 (6)	0.64 (0.42–0.98)	**0.038**	36 (35)	72 (8)	0.70 (0.58–1.45)	0.249
**High pERK1/2 or AR-515**	84 (37)	81 (5)			44 (42)	59 (8)		
**High pERK1/2 + AR-515**	86 (38)	90 (4)			24 (23)	79 (8)		

Supplementary Table 2: Relationship between ligand-independent AR phosphorylation and cancer-specific survival in patients with TNBC (*n* = 94)

	HER2-type (*n* = 29)				TNBC (*n* = 65)			
	N (%)	10yr-CSS % (SE)	Univariate HR (95% CI)	*P*	*N (%)*	10yr-CSS % (SE)	Univariate HR (95% CI)	*P*
**Total AR expression**								
**Low expression**	20 (69)	46 (12)	0.39 (0.09–1.79)	0.207	52 (80)	75 (6)	3.31 (1.35–8.11)	**0.006**
**High expression**	9 (31)	78 (14)			13 (20)	38 (13)		
**AR-515 phosphorylation**								
**Low expression**	17 (59)	44 (12)	0.30 (0.08–1.12)	0.056	26 (40)	76 (9)	1.70 (0.65–4.42)	0.274
**High expression**	12 (41)	74 (13)			39 (60)	62 (8)		
**ERK1/2 phosphorylation**								
**Low expression**	21 (72)	51 (12)	0.52 (0.11–2.40)	0.390	43 (66)	70 (7)	1.33 (0.54–3.25)	0.534
**High expression**	8 (28)	75 (15)			22 (34)	63 (11)		
**Combined AR + AR-515**								
**Low AR+AR-515**	13 (45)	42 (14)	0.40 (0.16–1.01)	0.114	23 (36)	77 (9)	2.19 (1.14–4.20)	**0.007**
**High AR or AR-515**	11 (38)	53 (15)			32 (49)	73 (8)		
**High AR + AR-515**	5 (17)	100 (0)			10 (15)	30 (14)		
**Combined pERK1/2 + AR-515**								
**Low pERK1/2+AR-515**	10 (36)	33 (16)	0.29 (0.09–0.90)	0.076	21 (32)	85 (8)	1.35 (0.77–2.34)	0.065
**High pERK1/2 or AR-515**	16 (57)	68 (12)			27 (42)	51 (10)		
**High pERK1/2 + AR-515**	2 (7)	100 (0)			17 (26)	70 (11)		
